# La plaie cranio-cérébrale chez l'oxycéphale: quelle précaution prendre pour le traiter?

**DOI:** 10.11604/pamj.2015.22.112.7366

**Published:** 2015-10-08

**Authors:** Loubna Rifi, Amina Barkat, Abdessamad El Ouahabi

**Affiliations:** 1 Department of Neurosurgery, Hôpital des Spécialités ONO, CHU de Rabat, Rabat, Morocco; 2 Mohamed V University, Rabat, Morocco; 3 Medical Department of Neonatology Reanimation, The Reference National Centre of Neonatology and Nutrition of Mother and Child, Sick Child Hospital CHU de Rabat, Rabat, Morocco

**Keywords:** Oxycéphalie, plaie cranio-cérébrale, craniosténoses, Oxycephaly, cranio-cerebral wound, craniosynostosis

## Abstract

L'oxycéphalie isolée est une forme non syndromique des craniosténoses d'apparition tardive, elle est responsable d'hypertension intracrânienne insidieuse pouvant aboutir, sinon traitée précocement, à la cécité et à la débilité mentale. En général méconnue, elle peut être révélée par tout phénomène pouvant décompenser l'hypertension intracrânienne latente. Nous présentons un cas rare d'oxycéphalie révélée par un traumatisme crânien. Un jeune garçon de cinq ans s'est présenté avec une tuméfaction pariétale droite non réductible suite à une plaie cranio-cérébrale opérée à deux reprises à l’âge de trois ans puis six mois plus tard. Le bilan neuroradiologique a objectivé une hernie cérébrale avec empreintes digitiformes diffuses et une fusion des sutures crâniennes. Un bilan ophtalmologique a montré un strabisme convergent droit et un fond d’œil normal. Une chirurgie d'expansion crâniennea été réalisée. L’évolution post-opératoire était favorable. L'oxycéphalie harmonieuse peut passer inaperçue et un traumatisme crânien même bénin peut la révéler.

## Introduction

Secondaire à une synostose prématurée des sutures coronales sagittale, l'oxycéphalie se définit par un front reculé et surtout basculé en arrière en continuité avec l'arête nasale. Le plus souvent étroit et surmonté par une saillie médiane située au niveau de la fontanelle bregmatique. Le massif facial est normal dans ce type de craniosténoses. Le plus souvent méconnue car d'apparition tardive mais elle est responsable d'hypertension intracrânienne (HIC) insidieuse, avec baisse de l'acuité visuelle pouvant aboutir à la cécité, des céphalées et un retard mental si elle n'est pas traitée à temps. Dans notre département nous avons traités 182 craniosténoses dont 15 oxycéphalies (8,2%) qui se sont tous présentés avec un syndrome d'HIC à un âge moyen de 54 mois. Après une recherche sur Pub Med, sciences Directes et Google Scholar, notre observation clinique est la première publication d'oxycéphalie révélée par une plaie cranio-cérébrale.

## Patient et observation

Il s'agit d'une observation clinique d'un enfant de sexe masculin âgé de cinq ans, quatrième d'une fratrie de quatre enfants, de niveau socio-économique moyen, habitant la région du pré-Rif le nord du Maroc, sans aucune notion de consanguinité, le père est âgé de 40 ans et la mère de 37 ans. La grossesse était bien suivie et l'accouchement médicalisé. L'enfant est bien vacciné selon le programme national d'immunisation, il a présenté depuis l’âge de un an un strabisme convergent sous correction. L'enfant selon la famille avait un développement psychomoteur normal. Il a été victime à l’âge de trois ans d'un accident domestique, réception d'un objet contendant sur la tête (un miroir) responsable d'une plaie cranio-cérébrale, qui a été opérée au centre hospitalier régional ou une craniectomie à os perdue a été réalisée avec découverte d'une plaie de la dure-mère qui a nécessité une plastie dure-mérienne de 4X4 cm à partir de la galea, mais les suites opératoires étaient marquées par la survenue d'une hernie cérébrale au niveau de l'endroit de la craniectomie, ceci a motivé une reprise opératoire six mois plus tard avec la mise en place d'une cranioplastie par de la résine acrylique. Cependant, l'hernie cérébrale a persistée et était responsable d'un préjudice esthétique gênant. Un bilan neuroradiologique a été réalisé et l'enfant a été transféré dans notre département qui est une référence nationale pour la neurochirurgie pédiatrique. L'examen clinique à son admission a trouvé un enfant conscient en bon état général, apyrétique et normo-tendu, l'examen des membres ne trouve aucune anomalie, l'examen cardio-vasculaire et pleuro-pulmonaires estnormal. L'examen neurologique n'a révélé aucun déficit neurologique sensitivomoteur, ses reflexes sont présents. L'aspect du crâne bien que harmonieux, montrait un front reculé en arrière en continuité avec l'arête nasale avec absence de l'angle fronto-nasal, le rebord orbitaire supérieur est reculé, le périmètre crânien est de 50 cm, soit moins une déviation standard pour l’âge. Localement on trouve une cicatrice opératoire propre, une tuméfaction pariétale battante de 10 cm de grand axe surmontée par un plan dur correspondant au matériel de la cranioplastie. L'examen ophtalmologique a montré une discrète exophtalmie (grade I) un nystagmus horizontal, une atteinte du VI à droite, une acuité visuelle non chiffrée vu que l'enfant n’était pas coopérant, le fond d’œil était normal. Les autres paires crâniennes étaient indemnes.

**Figure 1:**
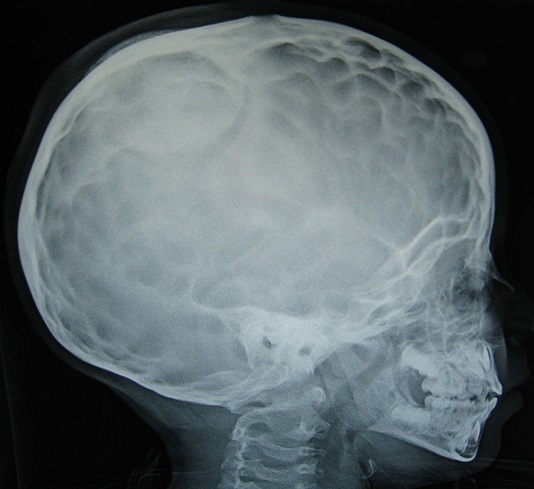
Radiographie du crâne simple de profil objective des empreintes digitiformes diffuses avec aspect d'ex-barrure pariétale et synostose des sutures coronales

**Figure 2:**
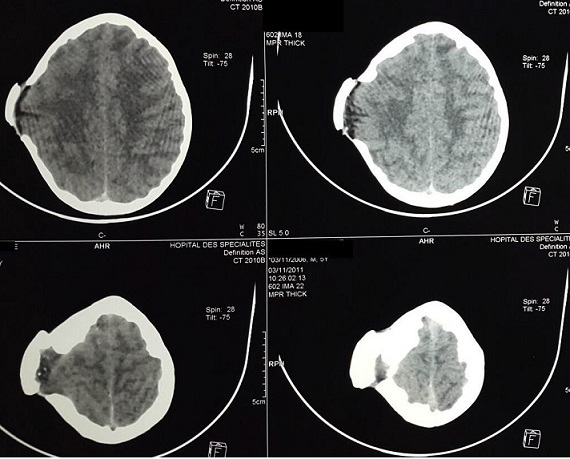
Radiographie du crâne simple de profil objective des empreintes digitiformes diffuses avec aspect d'ex-barrure pariétale et synostose des sutures coronales

**Figure 3:**
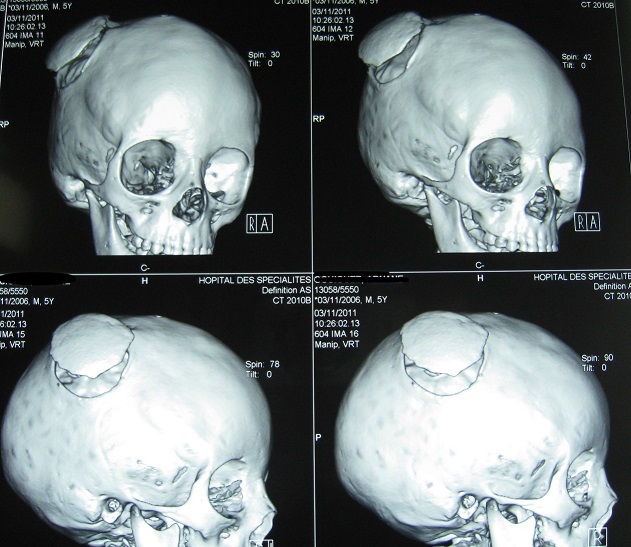
TDM en trois dimensions montre les empreintes digitiformes, une synostose coronales et sagittale avec un aspect harmonieux de la voûte et surtout la cranioplastie ex-barrée

**Figure 4:**
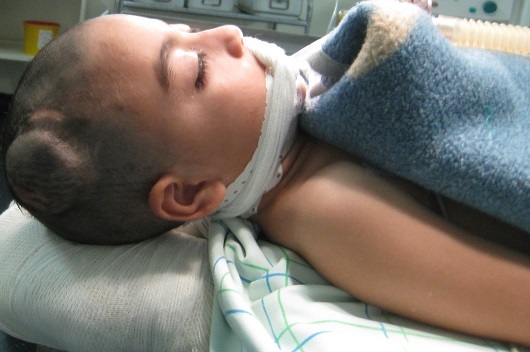
Montre l'aspect harmonieux de l'oxycéphalie, la position opératoire et la tuméfaction pariétale droite correspondant à l'ex-barrure du matériel de la cranioplastie

**Figure 5:**
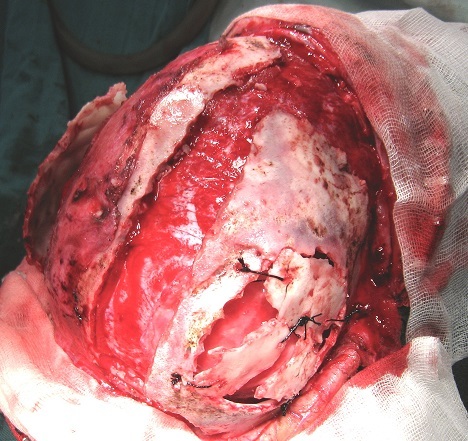
Peropératoire montrant la les volets bipariétaux avec la fermeture de la perte de substance osseuse par des bandes osseuses prélevées au niveau de la voûte

**Figure 6:**
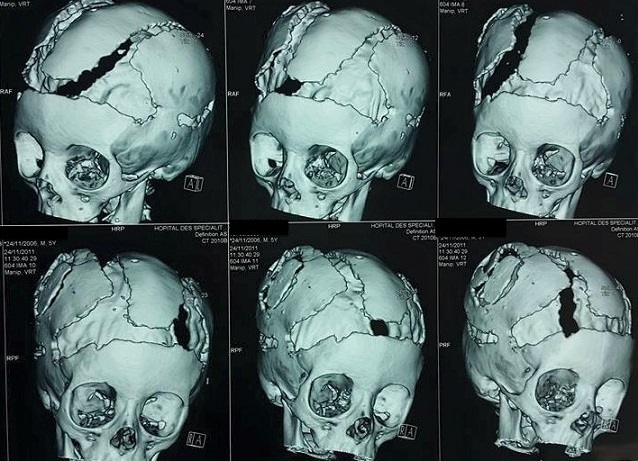
TDM en trois dimensions montrant la craniectomie décompressive et la fermeture de la perte de substance osseuse

**Figure 7:**
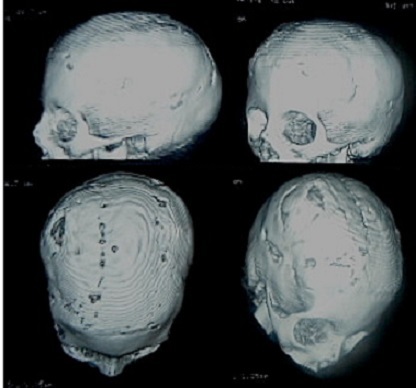
TDM en Trois dimensions réalisée 3 ans en post opératoire montre un aspect harmonieux avec une réossification correcte de la voûte sans empreintes digitiformes. Le défect osseux persistant ne nécessite aucun traitement chirurgical

Les fonctions supérieures étaient ralenties pour l’âge. L’étude électro-encéphale-graphique (EEG) a objectivée des bouffées généralisées en rapport avec une souffrance cérébrale diffuse mais le tracé du sommeil était bien organisé. Le bilan radiologique standard a montré des empreintes digitiformes diffuses et une synostose intéressant toutes les sutures du crâne avec un décollement de la cranioplastie (Figure 1). Le bilan scannographique en coupes axiales avec et sans injection du produit de contraste en fenêtres osseuses et parenchymateuses a montre une cranioplastie ex-barrée et une hernie parenchymateuses pariétale droite avec des empreintes digitiformes diffuses (Figure 2). La TDM en reconstruction trois dimensions a montre une synostose des sutures coronales et sagittale, les impressions digitiformes intéressant toute la voûte du crâne et a permis une meilleure définition de l'ex-barrure pariétale droite (Figure 3). Le bilan biologique préopératoire ne montrait aucune anomalie. Le taux d'hémoglobine était à 13g/l, l'hématocrite a 39%, et le temps de prothrombine est de 100%. L’échographie cardiaque et l’échographie abdomino-pelvienne ainsi que les radiologies des membres qu'on réalise de façon systématique devant toutes craniosténoses n'ont pas montrées d'autres malformations associées. Intervention chirurgicale: l'enfant a été opéré suite à une visite pré-anesthésique et une demande de deux culots globulaires, en décubitus dorsal, tête en flexion et dossier surélevé à 30^°^ par rapport à l'horizontal (Figure 4). Après une asepsie rigoureuse à la Bétadine et une infiltration à l'adrénaline+ xylocaïne à 1/400, on a démarré notre incision en zigzag à partir de l'ancienne incision cutanée, le lambeau cutané a été décollé progressivement avec hémostase soigneuse, on a disséqué la Galea avec précaution au pourtour de l'hernie cérébrale pariétale droite qui était recouverte par une cranioplastie en ciment acrylique, la plastie dure- mérienne recouvrant l'hernie cérébrale était indemne. On a réalisé deux volets pariéto-temporaux de part et d'autres de la ligne médiane réalisant. Le défect osseux de l'ancienne plaie cranio-cérébrale a été recouvert par des bandes osseuses prélevées au cours de la réalisation des volets pariéto- temporaux qui ont été fixées à l'os par du fils de soie 1-0 (Figure 5). On a réalisé une fermeture de laGalea par du fils résorbable et la peau était fermée en deux plans par du fils résorbable 3-0 sur un drain de Redon numéro 10 aspiratif. Les pertes sanguines étaient estimées à 100 cc en peropératoire, 30cc en post opératoire et une transfusion de 150 cc de culot globulaire a été réalisée. L'hémoglobine de contrôle était de 10,4 g/100ml et l'hématocrite à 31,2%. L'enfant a été mis sous antipyrétiques et antalgiques. L’évolution post opératoire immédiate était simple. Une TDM en trois dimensions post opératoire montre les résultats de la chirurgie décompressive (Figure 6). L’évolution après trois ans montre un très bon développement psychomoteur, l'enfant est actuellement scolarisé et réussi ses examens, son crâne ne présente pas d'anomalies. Le dernier examen ophtalmologique montre une acuité visuelle à 10/10 son strabisme est corrigé et l'examen du fond d’œil est normal. L'examen TDM coupes axiales avec la TDM en 3 dimensions montre une bonne ossification du crâne et absence d'empreintes digitiformes (Figure 7).

## Discussion

L'oxycéphalie isolée est une forme non syndromique de craniosténose tardive caractérisée par la fusion prématurée des sutures coronales et sagittales rarement les sutures coronales seules. Une croissance compensatrice dans la région de la fontanelle antérieure résulte en la formation d'un crâne pointu ou en forme de cône. Sa prévalence et son étiologie sont inconnues, elle n'est pas congénitale et n'est jamais diagnostiquée à la naissance. Décrite depuis l'antiquité et souvent confondues avec les brachycéphalies ou toutes craniosténoses multi-suturaires, c'est en 1977 que Mantaut et Striker ont décrit trois types d'oxycéphalie [1]: le type I acrocéphalie ou turricéphalie avec un front verticalisé et reculé; le type II l'oxycéphalie vraie avec un front reculé et basculé vers l'arrière et une saillie bregmatique responsable d'un crâne pointu; le type III l'oxycéphalie sans déformation mais avec un petit crâne réduit dans tous ses diamètres. Mais cette classification a été sujette à des critiques. Le type I correspond plus à une brachycéphalie dont l’âge de survenu et la prise en charge est complètement différent de l'oxycéphalie [2]. Le type II est le cas typique comme son nom étymologique indique «Oxus= pointu» est un crâne pointu reculé et basculé en arrière en continuité avec l'arête nasal et se termine par une bosse bregmatique. L'angle fronto-nasale est largement ouvert ce qui donne une impression d'exorbitisme. Le type III est une forme harmonieuse du type II qui correspond à notre cas clinique qui ne présente aucune dysmorphie visible. Sur le plan étiologique l'oxycéphalie est d'apparition tardive, elle n'est jamais diagnostiquée avant l’âge de trois ans ce qui la différencie des autres craniosténoses non syndromiques qui sont évidentes à la naissance. Marchac D et Renier ont eu la preuve d'un enfant avec radiographie du crâne normale à un an, avec un aspect typique d'oxycéphalie à 4 ans [3]. La cause de l'oxycéphalie n'est pas claire mais il a été démontré par Gault D et al [4] que le développement des craniosténoses chez l'enfant atteint de rachitisme était important ce qui a confirmé l’étude reportée par Reilly en 1964 qui a trouvé que le un tiers des enfants avec rachitisme développe une craniosténose. L'oxycéphalie serait plus fréquente sur le pourtour méditerranéen où elle représenterait 40 à 75% des craniosténoses alors qu'elle ne représente que 6 à 13% dans les séries nord américaines. Dans notre département sur 182 cas de craniosténoses opérés seulement 15 cas avaient une oxycéphalie ce qui représente uniquement 8,4%. La pression intracrânienne élevée est le signe clinique caractéristique de cette pathologie, qui conduit fréquemment à des complications ophtalmologiquesqui peuvent aboutir à la cécitéet à un déficit intellectuel modéré ou sévère [5]. La gravité de l'oxycéphalie est liée à l'HIC présente dans 61,6% des cas, un œdème papillaire dans 10% des cas et 13% ont une atrophie optique au moment du diagnostic [6]. Le facteur principal de cette HIC est la réduction du volume crânien qui peut être harmonieuse, sans dysmorphie visible. Cependant il faut insister sur la complète dissociation qui existe entre la pression intracrânienne et le fond d’œil, qui reste normal chez presque deux tiers des enfants qui ont une HIC malgré son caractère chronique. Ces constatations sont bien illustrées dans notre observation: l'enfant avait des signes d'HIC à l’âge de trois ans responsable d'une hernie cérébrale non corrigée par une cranioplastie et un strabisme convergent de l’œil droit sous correction mais son fond d’œil était normal, il était ralentit par rapport à son âge mais sa capacité à l'apprentissage était normale.

Le diagnostic est basé sur l'examen clinique, l'examen radiologique et la tomodensitométrie 3D du crâne. La radiographie du crâne de face et de profil dans notre observation a montré des empreintes digitiformes avec saillie de la cranioplastie, le bilan scannographique en coupes axiales a confirmé l'hernie cérébrale et la TDM en trois dimensions a montrée une soudure des sutures coronales et sagittale avec les empreintes digitiformes diffuses et l'exclusion de la cranioplastie. L'absence d'anomalies faciales et/ou des membres permet de différencier l'oxycéphalie isolée des autres brachycéphalies syndromiques. Les radiographies des extrémités et les échographies cardiaques et abdominopelviennes étaient sans aucune anomalie dans notre cas clinique. On n'a pas décelé d'autres anomalies cérébrales ou viscérales chez notre patient. L’étude électro-encephalo-graphique (EEG) a révélée des signes en faveur de souffrance cérébrale diffuse avec un tracé de sommeil bien organisé, témoignant d'une HIC présente et insidieuse. Le traitement chirurgical des oxycéphalies est un sujet de débat, en fonction du but de la chirurgie, l’âge idéal pour l'intervention, la technique chirurgicale et les résultats morphologiques [7]. La chirurgie d'expansion crânienne est essentielle pour soulager la pression intracrânienne et assurer un niveau intellectuel satisfaisant. Elle doit être effectuée durant l'enfance, dès que le diagnostic clinique est établi. De nombreuses techniques chirurgicales des craniectomies linéaires ou suturotomies et des fragmentations de la voûte ont été publiées et qui avaient comme objectif principal de décomprimer l'encéphale sans changer la forme du crâne ses techniques ont été abandonnées car elles exposaient à des récidives où elles compromettaient la réossification. Les techniques cranio-faciales ce sont développées suite aux travaux de Rougerie et al en 1972 [8]. Ses techniques permettent une expansion et une reconstruction crânienne, elles corrigent en même temps l'HIC et la dysmorphie. Dans notre observation vu l'absence de dysmorphie la réalisation de volets pariéto-temporaux avec une fermeture de la perte de substance osseuse par des fragments d'os prélevés sur le site même de la craniectomie ont permis de faire une correction définitive. En absence de traitement approprié, l'HIC insidieuse aboutit au déficit intellectuel parfois définitif et aucune récupération ne peutêtre notée en post opératoire. Cependant l’œdème papillaire qui est la conséquence d'une pression intracrânienne élevée, disparaît habituellement en post-opératoire. Après la chirurgie, la pression intracrânienne retourne à la normale et le déficit intellectuel se stabilise dans la plupart des cas. C'est le cas notre observation ou la hernie cérébrale a disparue avec une ré-ossification harmonieuse, l'enfant a un développement intellectuel normal pour son âge dans la mesure ou il suit une scolarité normale et réussi ses examens.

## Conclusion

L'oxycéphalie est une entité rare et grave car elle peut passer inaperçue exposant à des risques d'HIC chronique et insidieuse pouvant aboutir à la cécité ou la débilité mentale si elle n'est pas diagnostiquée à temps. Sa décompensation brutale peut survenir à la suite d'un traumatisme crânien c'est ce qui a été illustré par notre cas clinique, ou à la suite d'une affection intercurrente.
